# Serum corin is associated with the risk of chronic heart failure

**DOI:** 10.18632/oncotarget.22227

**Published:** 2017-11-01

**Authors:** Zongliang Yu, Xiang Lu, Weiting Xu, Mengchao Jin, Yifei Tao, Xiang Zhou

**Affiliations:** ^1^ Department of Cardiology, The First People's Hospital of Kunshan Affiliated to Jiangsu University, Kunshan, China; ^2^ Department of Geriatrics, Sir Run Run Hospital, Nanjing Medical University, Nanjing, China; ^3^ Department of Cardiology, The Second Affiliated Hospital of Soochow University, Suzhou, China

**Keywords:** chronic heart failure, corin, risk

## Abstract

It has been well documented that corin is a critical protease involved in the regulation of blood pressure and cardiac function. We performed a case-control study to determine whether serum corin is associated with the risk of chronic heart failure (CHF). We included 484 consecutive CHF patients and 484 control subjects to investigate the potential relationship between serum corin and CHF using logistic regression analysis. Compared with healthy controls, the CHF patients were more likely to have histories of hypertension and diabetes, and had higher levels of N-terminal pro-brain natriuretic peptide and lower levels of corin. The odds ratios of ischemic and non-ischemic HF were significantly reduced with the growing levels of serum corin after multivariate adjustment. Moreover, the decrease in serum corin levels seemed to be associated with the severity of CHF. In conclusion, our study suggested that serum corin levels were reduced in CHF patients and inversely correlated with the incidence of ischemic and non-ischemic HF.

## INTRODUCTION

Corin is known as a transmembrane serine protease and has multiple physiological functions in the cardiovascular system. This protease mainly exists in myocardial cells and plays crucial roles in processing pro-natriuretic peptides, thereby regulating salt-water balance and vasodilation [1]. Recently, increasing evidence has indicated that corin is actively involved in the modulation of blood pressure and cardiac function. It was reported that corin knockout eventually resulted in spontaneous hypertension and myocardial dysfunction in mice [2]. On the contrary, overexpression of corin was found to improve cardiac function and survival in mice with dilated cardiomyopathy [3]. Moreover, in population-based epidemiological studies, serum corin levels were found to be decreased in patients with acute myocardial infarction and stoke [4, 5].

Chronic heart failure (CHF), a clinical syndrome characterized by reduced myocardial contractility, hemodynamic abnormality and neuroendocrine activation, is the major cause of mortality and morbidity in patients with cardiovascular diseases [6]. Over the past few years, numerous risk factors have been identified to be associated with the development of CHF. In the present study, we carried out a hospital-based case-control study to investigate the potential relationship between serum corin and CHF risk.

## RESULTS

In this study, there were 484 control subjects and 484 CHF patients, including 235 coronary heart disease, 97 valvular heart disease, 83 hypertensive heart disease, 42 dilated cardiomyopathy and 27 other cardiovascular diseases. The baseline characteristics of the study population are shown in Tables 1 and 2. The serum levels of corin were significantly decreased in CHF patients compared with those in the controls. The CHF patients were more likely to have histories of hypertension and diabetes, and had lower eGFR and higher NT-proBNP levels. In addition, the CHF patients were divided into 2 groups according to myocardial ischemia. Patients with ischemic HF tended to be male and older, and were more likely to have histories of hypertension, diabetes, hyperlipidemia and smoking.

**Table 1:** Baseline characteristics of CHF patients and controls

	Control (n = 484)	CHF (n = 484)	P value
Age (years)	62.8 ± 7.4	64.5 ± 8.3	NS
Male (%)	292 (60.3)	306 (63.2)	NS
Hypertension (%)	216 (44.6)	278 (57.4)	< 0.001
Diabetes (%)	90 (18.6)	142 (29.3)	< 0.001
Hyperlipidemia (%)	207 (42.8)	221 (45.7)	NS
Smoking (%)	185 (38.2)	203 (41.9)	NS
eGFR (ml/min/1.73 m^2^)	89.4 ± 8.3	67.8 ± 10.2	< 0.001
NT-proBNP (pg/ml)	271 ± 35	3054 ± 420	< 0.001
Corin (pg/ml)	1182 ± 237	763 ± 175	< 0.001

Data are expressed as mean ± SD or numbers (percentage). CHF = chronic heart failure; eGFR = estimated glomerular filtration rate; NT-proBNP = N-terminal pro-brain natriuretic peptide.

**Table 2:** Characteristics of patients subdivided by ischemic and non-ischemic HF

	Ischemic (n = 235)	Non-ischemic (n = 249)	P value
Age (years)	68.2 ± 7.6	61.0 ± 8.9	< 0.001
Male (%)	165 (70.2)	141 (56.6)	< 0.001
Hypertension (%)	153 (65.1)	125 (50.2)	< 0.001
Diabetes (%)	82 (34.9)	60 (24.1)	< 0.001
Hyperlipidemia (%)	125 (53.2)	96 (38.6)	< 0.001
Smoking (%)	110 (46.8)	93 (37.3)	< 0.001
eGFR (ml/min/1.73 m^2^)	70.3 ± 9.8	65.4 ± 10.7	NS
NT-proBNP (pg/ml)	2936 ± 395	3165 ± 452	NS
Corin (pg/ml)	809 ± 194	720 ± 158	NS

Data are expressed as mean ± SD or numbers (percentage). HF = heart failure; eGFR = estimated glomerular filtration rate; NT-proBNP = N-terminal pro-brain natriuretic peptide.

Participants were divided into quartiles of corin levels in control subjects to assess the association between serum corin and CHF. With the lowest quartile as a reference, the ORs of CHF were calculated and the results are presented in Table 3. Our findings indicated that CHF was significantly correlated with reduced levels of corin (*P* for trend, < 0.001) after adjusting for hypertension, diabetes, eGFR and NT-proBNP.

**Table 3:** Association of serum corin with CHF

Corin (quartiles)	Control (n = 484)	CHF (n = 484)	Adjusted OR^*^ (95% CI)
Q1 (< 813 pg/ml)	121	186	1.00
Q2 (813-1129 pg/ml)	121	150	0.83 (0.59-1.12)
Q3 (1129-1378 pg/ml)	121	97	0.56 (0.37-0.85)
Q4 (> 1378 pg/ml)	121	51	0.31 (0.18-0.64)
*P* for trend			< 0.001

^*^Adjustment for hypertension, diabetes, eGFR and NT-proBNP in analysis. CHF = chronic heart failure; OR = odds ratio; CI = confidence interval.

As shown in Table 4, both ischemic and non-ischemic HF were associated with the decreasing levels of serum corin after multivariate adjustment (P for trend, < 0.001). Participants in the highest quartile had 0.37× (OR, 0.37; 95% CI, 0.20-0.71) the OR of ischemic HF and 0.26× (OR, 0.26; 95% CI, 0.15-0.58) the OR of non-ischemic HF for participants in the lowest quartile.

**Table 4:** Association of serum corin with ischemic and non-ischemic HF

Corin (quartiles)	Ischemic (n = 235)		Non-ischemic (n = 249)	
	Case/control	Adjusted OR^*^ (95% CI)	Case/control	Adjusted OR^*^ (95% CI)
Q1 (< 813 pg/ml)	86/121	1.00	100/121	1.00
Q2 (813-1129 pg/ml)	72/121	0.86 (0.57-1.23)	78/121	0.80 (0.61-1.09)
Q3 (1129-1378 pg/ml)	48/121	0.59 (0.34-0.92)	49/121	0.52 (0.29-0.83)
Q4 (> 1378 pg/ml)	29/121	0.37 (0.20-0.71)	22/121	0.26 (0.15-0.58)
*P* for trend		< 0.001		< 0.001

^*^Adjustment for hypertension, diabetes, eGFR and NT-proBNP in analysis. HF = heart failure; OR = odds ratio; CI = confidence interval.

**Figure 1:**
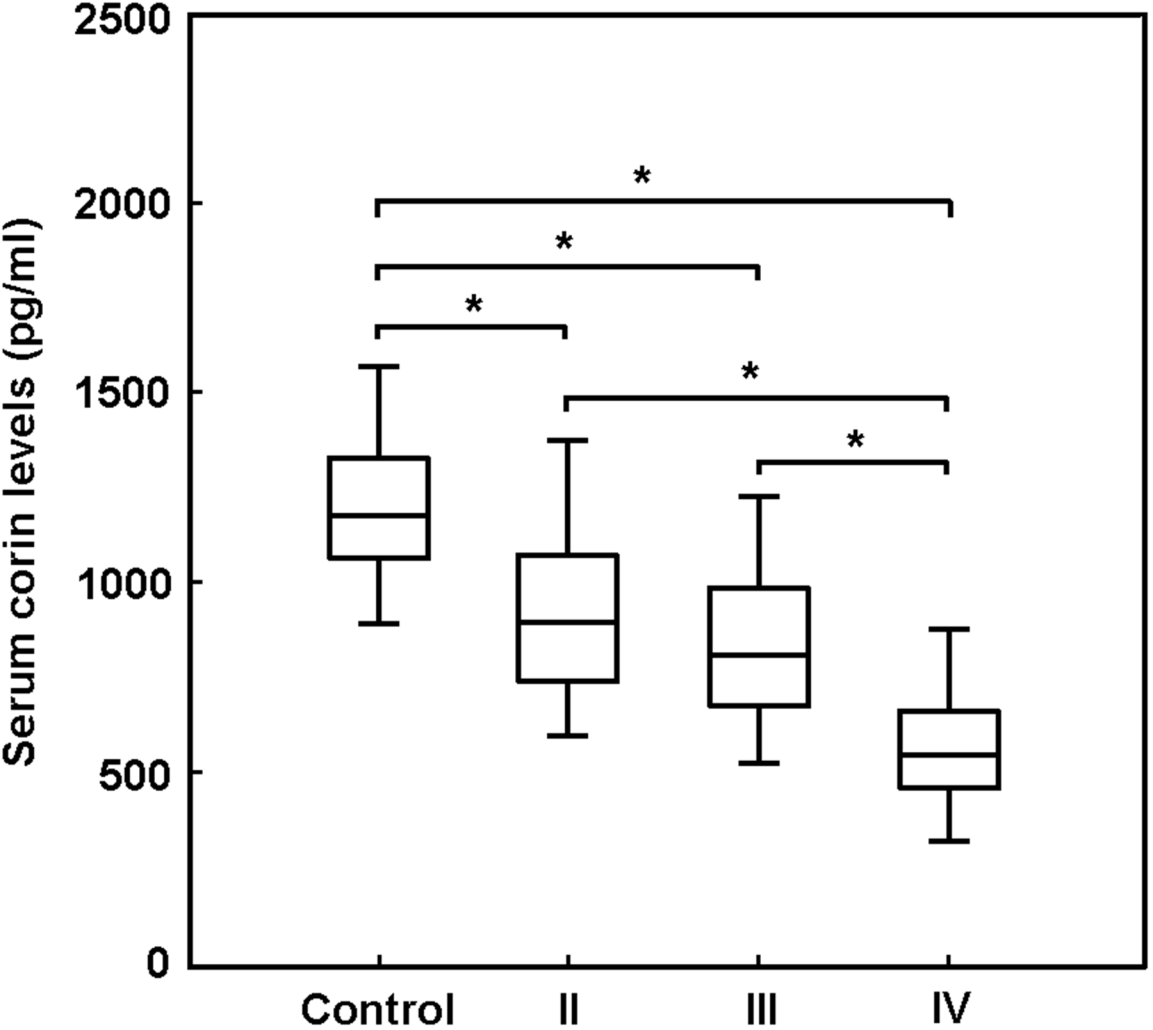
The cardiac function in patients with chronic heart failure (CHF) was divided into 4 groups according to the NYHA classification The serum levels of corin were progressively lower in CHF patients with more severe cardiac dysfunction. ^*^ P < 0.05.

Our findings revealed that the decrease in serum corin levels seemed to be associated with the severity of CHF. The cardiac function in CHF patients was divided into 4 groups according to the NYHA classification. As shown in Figure 1, the serum levels of corin were progressively lower in CHF patients with more severe cardiac dysfunction.

## DISCUSSION

We carried out this case-control study to determine the association of serum corin with CHF risk in a Chinese population. Our results showed that serum levels of corin were significantly decreased in CHF patients. The ORs of ischemic and non-ischemic HF were markedly reduced with the growing levels of serum corin following multivariate adjustment, which suggests that serum corin was inversely correlated with the incidence of CHF.

Corin, which belongs to the transmembrane serine protease family, has a cytoplasmic tail and a single-span transmembrane domain near the N-terminus [7]. Accumulating evidence has demonstrated that corin is involved in the regulation of blood pressure and cardiac function by activating natriuretic peptides. Under the conditions of volume or pressure overload, there is increased production of natriuretic peptides in the heart, which consequently induces diuresis and vasodilation [8]. Recent studies by Zhou et al. have revealed that corin is a valuable prognostic marker of major adverse cardiovascular events in patients with CHF and acute myocardial infarction, independent of conventional risk factors [9, 10]. In the present study, serum corin levels were found to be reduced in CHF patients, which was in accordance with the results of a previous study [11]. In addition, our findings also indicated that serum corin was inversely associated with the incidence of ischemic and non-ischemic HF. Furthermore, serum corin levels were progressively lower in CHF patients with more severe cardiac dysfunction.

This study has several limitations. Firstly, it remains unknown whether reduced corin is the cause or the result of CHF, so the conclusions need to be further verified in prospective cohort studies. Secondly, this study was conducted in a Chinese population and the conclusions should be cautiously extrapolated to other ethnic groups. Thirdly, we cannot avoid selection bias, information bias and confounding bias in this study.

In summary, our study demonstrated that serum corin levels were decreased in CHF patients and inversely correlated with the incidence of CHF.

## MATERIALS AND METHODS

### Study population

We consecutively recruited 484 CHF patients with different causes including ischemic, valvular, hypertensive, and cardiomyopathic etiologies between September 2015 and May 2017. The diagnosis of CHF was based on typical symptoms and signs and evidence of left ventricular enlargement and systolic functional impairment by echocardiography. A total of 484 control subjects were randomly selected from the healthy participants. This study was approved by the ethics committee of Soochow University and written informed consents were obtained from all participants.

### Clinical and biochemical measurements

The clinical and biochemical data of patients were obtained from medical records. Hypertension was defined as systolic blood pressure ≥ 140 mmHg or diastolic blood pressure ≥ 90 mmHg or receiving antihypertensive therapy. Diabetes was defined as fasting glucose ≥ 7.0 mmol/L or 2-hour postprandial blood glucose ≥ 11.1 mmol/L or use of antidiabetic agents. Hyperlipidemia was defined as triglyceride concentration > 1.7 mmol/L or total cholesterol concentration > 5.7 mmol/L or use of lipid-regulating drugs. The estimated glomerular filtration rate (eGFR) was calculated using the MDRD study equation, taking into account gender, age, race, and serum creatinine. Serum levels of corin and N-terminal pro-brain natriuretic peptide (NT-proBNP) were measured using enzyme-linked immunosorbent assay kits (R&D Systems, Minneapolis, USA; Roche Diagnostics, Mannheim, Germany) according to the manufacturer's instructions.

### Statistical analysis

Data are presented as mean ± SD or percentages. All statistical analyses were performed using SPSS software. Continuous variables were analyzed by Student's t test, while categorical variables were compared by χ^2^ test. Odds ratio (OR) and 95% confidence interval (CI) were calculated to determine the association between serum corin and CHF risk. Multiple logistic regression analysis was conducted to adjust for hypertension, diabetes, eGFR and NT-proBNP. A *P* value < 0.05 was considered statistically significant in this study.
